# Classical Cerebrospinal Fluid Biomarkers in Dementia with Lewy Bodies

**DOI:** 10.3390/medicina58050612

**Published:** 2022-04-28

**Authors:** Aikaterini Foska, Ioanna Tsantzali, Eleni Sideri, Maria Ioanna Stefanou, Eleni Bakola, Dimitrios K. Kitsos, Christina Zompola, Anastasios Bonakis, Sotirios Giannopoulos, Konstantinos I. Voumvourakis, Georgios Tsivgoulis, George P. Paraskevas

**Affiliations:** 2nd Department of Neurology, School of Medicine, National and Kapodistrian University of Athens, “Attikon” General University Hospital, 12462 Athens, Greece; dkfoska@gmail.com (A.F.); docjo1989@gmail.com (I.T.); elenisideri1985@gmail.com (E.S.); marianna421@hotmail.co.uk (M.I.S.); elbakola@yahoo.gr (E.B.); dkitsos@icloud.com (D.K.K.); chriszompola@yahoo.gr (C.Z.); bonakistasos@med.uoa.gr (A.B.); sgiannop@uoi.gr (S.G.); cvoumvou@otenet.gr (K.I.V.); tsivgoulisgiorg@yahoo.gr (G.T.)

**Keywords:** Dementia with Lewy bodies, Alzheimer’s disease, cerebrospinal fluid, biomarkers, tau, phospho-tau, beta-amyloid, a-synuclein

## Abstract

The use and interpretation of diagnostic cerebrospinal fluid (CSF) biomarkers for neurodegenerative disorders, such as Dementia with Lewy bodies (DLB), represent a clinical challenge. According to the literature, the composition of CSF in DLB patients varies. Some patients present with reduced levels of amyloid, others with full Alzheimer Disease CSF profile (both reduced amyloid and increased phospho-tau) and some with a normal profile. Some patients may present with abnormal levels of a-synuclein. Continuous efforts will be required to establish useful CSF biomarkers for the early diagnosis of DLB. Given the heterogeneity of methods and results between studies, further validation is fundamental before conclusions can be drawn.

## 1. Introduction

Dementia with Lewy bodies (DLB) is the second most common cause of neurodegenerative dementia in the elderly, following Alzheimer’s disease, and it is characterized by a progressive cognitive deterioration, accompanied by various combinations of clinical features, including parkinsonism, visual hallucinations, fluctuations (alterations in cognition, attention, arousal, alertness) psychiatric/behavioral features, REM sleep behavior disorder and autonomic dysfunction [1]. Furthermore, there are some additional clinical features that are commonly present and may persist over time, such as hypersomnia, hyposmia and transient episodes of unresponsiveness, the latter representing an extreme form of cognitive fluctuation, sometimes difficult to distinguish from true syncope or severe neuroleptic sensitivity. The pathologic hallmarks of DLB are the Lewy bodies, which are clumps of abnormal protein particles that accumulate in the brain within neurons and the key protein involved is polymerized α-synuclein (α-syn), a member of a conserved family of proteins that also includes β-synuclein and γ-synuclein, and was originally described as the precursor protein for the non-amyloid component of Alzheimer’s disease senile plaques [2]. However, there is increasing evidence that lipid membrane fragments and distorted organelles, together with a non-fibrillar form of α-Syn, are also significant structural building blocks for the formation of Lewy bodies [3]. In addition, α-Syn seeding-competent aggregates, oligomers and fibrils are found in the biological fluids, such as cerebrospinal fluid (CSF) and plasma of patients with synucleinopathies, and their seeding activity and structural properties show promising diagnostic potential [4].

Established (classical) biomarkers of dementia in the cerebrospinal fluid (CSF), namely amyloid beta with 42 amino acids (Aβ_42_), a marker of amyloid pathology (plaque formation), total tau protein (τ_T_), a non-specific marker of neurodegeneration and neuronal/axonal loss and phosphorylated tau protein at threonine 181 (τ_P-181_), a marker of tau pathology (neurofibrillary tangle formation) have been proven useful in the (differential) diagnosis of Alzheimer’s disease (AD) and have been incorporated in AD diagnostic criteria and guidelines [5]. The latter provide a useful aid, based on specific AD clinical phenotypes, together with in-vivo evidence of Alzheimer’s pathology through either a molecular AD signature in the CSF or positive amyloid imaging [5]. The typical CSF profile for AD is characterized by low Aβ_42_ and high τ_P-181_, based on the so-called AT(N) system [6], which introduced the concept of theorizing Alzheimer’s disease as a biological process, regardless of the presence of typical or atypical symptoms and the stage of the disease (dementia, mild cognitive impairment or even preclinical). However, for DLB, the situation remains unclear.

## 2. CSF Biomarkers in Dementia with Lewy Bodies

The development of widely applicable CSF biomarkers for DLB remains unclear. To date, the three classical biomarkers Aβ_42_, τ_P-181_ and τ_T_ may be more useful in determining the concomitant pathology of AD or in predicting cognitive impairment. On the other hand, CSF α-synuclein has not yet been shown to be an established biomarker but appears to be a promising one.

### 2.1. Reduction in Aβ_42_

A typical finding in DLB patients is a decrease in CSF Aβ_42_ levels, and this may be more common than previously thought. Aβ is a proteolytic cleavage product of amyloid precursor protein (APP). Decreased Aβ_42_ in the CSF is associated with neuropathological features in the brain, including amyloid plaques and neuronal loss, findings that have been proposed to be good predictors of cognitive impairment and dementia detection [7]. Several studies reported these reduced levels of Aβ_42_ in DLB, especially at the demented stage [7,8,9,10]. In the first mentioned study, a total of 166 CSF samples were collected at the memory clinic of Strasbourg. They were obtained from prodromal DLB (pro-DLB), DLB dementia, prodromal AD (pro-AD), and AD dementia patients, and elderly controls (phospho-Tau181, total-Tau, Aβ_42_, and Aβ_40_). At the prodromal stage, contrary to AD patients, DLB patients’ biomarker levels in the CSF were not altered. At the demented stage of DLB, A42 levels were reduced [8]. In a large cohort study, in which 375 DLB patients, 164 Parkinson’s disease (PD) patients without dementia, and 55 PD patients with dementia (PDD) from 10 centers were included, and it was noted that patients with reduced Aβ_42_ were older, more often females, had a shorter duration of the disease and had severe cognitive impairment [10]. It has been observed that DLB is characterized by reductions in Aβ_38_, Aβ_40_, and Aβ_42_, while AD is characterized by a relatively isolated reduction in Aβ_42_. This study included 72 patients with a diagnosis of probable DLB and matched them for age and sex, with 38 patients with a diagnosis of probable AD and 38 subjects with subjective cognitive decline who served as controls. The abovementioned patients and controls were selected from the Amsterdam Dementia Cohort [11]. The reductions in all three CSF Aβ peptides are independent of co-morbid AD pathology or APOE genotype, suggesting that Aβ metabolism is affected in DLB, even in the absence of co-morbid AD pathology, and that different pathogenic biological processes may be involved in Aβ-peptide-related amyloidogenesis in DLB versus AD [11]. Reduced levels of Aβ_42_ may be associated with more rapid cognitive decline in DLB patients, as was concluded by a European multicenter study on DLB, in which they selected 100 patients with diagnostic criteria for probable DLB [12]. A retrospective study conducted during a 3-year period in Greece revealed that this reduction is more common than expected, exceeding 80% of DLB patients and, in almost half of them, Aβ_42_ was the only abnormal biomarker [13]. However, other studies suggest that Aβ_42_ could show less prominent or marginal reduction or may even be increased in DLB as compared to AD, as proposed by a study with 1194 patients diagnosed with several neurodegenerative diseases, and another, in which they include CSF samples of 33 patients with probable AD without parkinsonism, 25 patients with all the core features of DLB, and 46 age-matched controls [14,15]. With more details and more precision, Bousiges et al. conducted a study involving patients diagnosed upon clinical and imaging data with prodromal DLB, DLB dementia, prodromal AD, AD dementia patients, and elderly controls, while autopsy verification was not carried out [8]. Inger van Steenoven et al., in 2016, conducted a large multicenter cohort study, which included 375 DLB patients, 164 Parkinson’s disease patients without dementia, and 55 PD patients with dementia without the use of a control group. The study population had a clinical diagnosis of probable DLB, PDD, or PD with available CSF biomarker data from 10 participating centers (academic memory clinics and movement disorder clinics) in eight countries [10]. Inger van Steenoven et al., in 2019, included 72 patients with a diagnosis of probable DLB and matched them for age and sex with 38 patients with a diagnosis of probable AD and 38 subjects with subjective cognitive decline who served as controls. Patients and controls were selected from the Amsterdam Dementia Cohort, and were assessed at the Alzheimer Center Amsterdam, between January 2000 and December 2017, based on the availability of CSF. All selected patients underwent an extensive standardized clinical and imaging workup [11]. Abdelnour et al., in 2019, selected 100 patients with diagnostic criteria for probable DLB from a European multicenter study of DLB. The diagnosis of DLB was made according to the consensus criteria by the treating physician, a group of at least two expert clinicians, or by a multidisciplinary team at a consensus diagnostic meeting, on the basis of all available clinical and diagnostic test data [12]. The retrospective study of Paraskevas et al., in 2019, consisted of all consecutive patients examined in their department during a 3-year period meeting the diagnostic criteria of probable DLB, according to the fourth consensus report of the DLB consortium and had CSF biomarker determination [13]. Some of the aforementioned studies were large multicenter studies, while some were smaller cohort studies, some of them lacked control group, while others included groups with other neurodegenerative diseases and clinical evaluation—neuropsychological testing differed among the various studies. The discrepancies between the above studies emphasize the importance of strict adherence to harmonization protocols and guidelines for pre-analytical handling of samples, biochemical (analytical) procedures, and correct clinical evaluation of patients.

### 2.2. Typical CSF AD Biomarker Profile

In a large memory clinic cohort [14] and in large multicenter studies in 2016 [8,10], it was observed that 25–32% of the DLB patients had CSF profiles compatible with AD, as defined by pathological Aβ_42_ combined with pathological τ_Τ_ and/or τ_P-181_. In line with the previous observation, another study, in 2019 [13], revealed that almost 40% of the included patients may show the typical CSF AD biomarker. Only a small percentage of DLB patients present with all three classical biomarkers normal or a non-specific increase in τ_Τ_ [13].

Thus, a reduction in Aβ_42_ is a rather frequent finding in patients with DLB. However, a significant percentage may show the typical CSF AD profile, indicating mixed pathology, not only with amyloid plaques but also with tangles, compatible with the additional presence of AD. Indeed, there seems to be a spectrum with pure DLB and pure AD at the two ends and mixed cases in between [16]. This concomitant AD biochemistry/pathology might not occur from disease onset. Since the presence of AD concomitant pathology increases with age or disease duration [17,18], one may hypothesize that AD and DLB patients share important common underlying molecular mechanisms and, while DLB starts as synucleinopathy, with disease progression, it may gradually enter the Alzheimer continuum in some patients (Table 1). Τhose last studies were conducted in the USA, consisting of a large number of patients. Clarifying Aβ metabolism is vital to understanding Aβ-peptide-related amyloidogenesis in DLB and could lead to new therapeutic approaches.

### 2.3. α-Synuclein

Although α-syn is not a classical biomarker, it is important to study its possible diagnostic role. Intracellular accumulation of α-syn, a protein that is abundantly expressed in the brain, is a feature of several late-onset synucleinopathies, such as DLB [2,3]. Similarly neurofibrillary tangles and amyloid plaques are the typical pathological findings of AD. It has been shown that α-syn is present in detectable amounts in CSF of normal subjects and Parkinson’s disease patients, with its origin mostly brain-derived and, thus, although not yet an established (classical) biomarker, it has received much attention as a candidate biomarker in synucleinopathies.

In a patient with a clinical presentation suggestive of DLB, positive CSF results for AD biomarkers indicate the presence of AD and may be compatible with mixed pathology (both synucleinopathy and AD) [19,20,21]. The recognition of multiple pathologies underlying dementia and the identification of the features that could predict the Lewy Body pathology in AD patients was the objective of those studies. However, the presence of AD alone presenting with atypical features resembling DLB has been described [22]. Since DLB is a synucleinopathy, quantitation of α-syn could be useful in the differential diagnosis between DLB and mixed cases. However, results so far are conflicting [23]. A detection of decreased CSF α-syn was observed by a cohort study in 2011 [24], whereas a publication in 2013 [25] revealed a significant increase in CSF α-syn levels in DLB patients, as compared to AD groups and normal controls. On the contrary, in 2016, another published study [26] showed CSF levels of total α-syn to be lower in DLB and PD compared to controls and AD. A cross-sectional study [27], conducted in 2018, revealed that higher levels of α-Syn could differentiate DLB from PDD and AD patients.

The form of quantitated α-syn may play some role, since synucleinopathies, such as DLB, may exhibit lower total α-syn and higher ratio of phosphorylated/total α-syn ratiο, as compared to other neurodegenerative diseases [28]. Furthermore, α-syn determination by Real-Time Quaking-Induced Conversion Assay (RT-QuIC) may prove a robust biomarker for prodromal DLB, as shown by a recent research team [29], but still, well-designed studies with a large number of participants need to be conducted.

The heterogeneity of results on α-syn could have been attributed, at least to some degree, to confounding factors. Initially, the immunoassays used are based on different antibodies that recognize different fragments of the protein and with variable affinity. In addition, patient cohorts show high variability at the time of CSF collection. Further, the absence of the application of strict standardized instructions regarding the collection and storage protocols and the limitation of the presence of blood in the CSF sample are significant hurdles. This suggests that CSF α-syn could serve as a potential marker of synucleinopathy if only a number of the above-mentioned factors have been controlled to reduce the observed variability in results. Furthermore, there should be application of standard instructions on collection and storage protocols and also for blood contamination, since the presence of blood in CSF alters the results. Among the various confounders of CNS α-Syn quantification, blood contamination has been widely recognized, as α-Syn is abundant in the red blood cells. Nevertheless, other methodological differences should be kept in mind, including differences in α-syn species quantified (its type may be different among the synucleinopathies) and, in addition, differences in capture/detection antibodies among the immunoassays used in different studies [30].

Furthermore, worth noting is that recent findings have pointed out the potential role of the two members of the family of synucleins {α-synuclein (140 amino acids) and β-synuclein (134 amino acids) as biomarkers for the diagnostic characterization of patients affected by synucleinopathies as DLB [31].

## 3. Discussion

Until now, classical (established) CSF biomarkers have included Aβ_42_, τ_P-181_ and τ_Τ_. The first biomarker in the AT(N) classification system (NIA-AA 2018) [6] is amyloid Aβ_42_, with reduced values being abnormal. Reduction in this biomarker alone may indicate (but not always) the Alzheimer’s *continuum* [19]. When τ_P-181_ is increased, in addition to decreased Aβ_42_, this is indicative of Alzheimer’s *disease*. DLB often overlaps with AD in clinical and pathological features, sometimes making it challenging to differentiate between these conditions. Some DLB patients may have abnormal (decreased) Aβ_42_ alone, while others may have both Aβ_42_ and τ_P-181_ abnormal. Taken together, the data from the literature described above indicate that decreased levels of CSF Aβ_42_ are a rather common finding in DLB patients, whereas a simultaneous increase in τ_P-181_ may be observed in a significant percentage of patients as the disease progresses [10,11,13]. The presence of a typical AD profile indicates that AD co-pathology in DLB might imply an evolving process. It is tempting to assume that the absence of the AD profile at one particular time does not preclude the development of such a profile during disease progression. Recently, it has been suggested that plasma τ_P-181_ may identify additional AD pathology in patients with DLB [32]. By avoiding lumbar puncture for CSF sampling, this could open new perspectives towards a less invasive diagnostic approach.

Therefore, given the fact that patients with DLB may frequently show a reduced Aβ_42_ or a typical AD profile, the presence of another more indicative biomarker is mandatory. This role could be attributed to *α*-syn. Nevertheless, there are still some methodological issues, currently preventing CSF *α*-syn to be an established biomarker, although it may become so in the near future. This could be feasible by achieving some control of several confounding parameters, in addition to the standardization of *α*-syn species measured.

Furthermore, given the overlapping of neuropathological, neurochemical and neuropsychiatric profiles of neurodegenerative diseases, larger studies of homogeneous design are required to demonstrate the relationship of the CSF biomarkers and the underlying pathology.

## 4. Conclusions

Recognizing DLB remains challenging due to the highly variable presentation of clinical symptoms, which include cognitive fluctuations, visual hallucinations, parkinsonism, sleep disorders and autonomic dysfunction, and the fact there is considerable clinical and pathological overlap, primarily with AD. They may occur together, not uncommonly, especially in older patients, but correct clinical identification of the underlying pathology is not always easy. The discovery of validated biomarkers for DLB is essential for the early diagnosis and treatment of patients affected by this prominent cause of neurodegenerative dementia. Currently, the only condition recognized by established CSF biomarkers is the presence or absence of concomitant AD. Diagnosis “by inclusion” has not been achieved yet. Until now, quantitative analysis of CSF α-syn levels has varied widely between studies, probably reflecting (pre)analytical issues, whilst a real biological cause cannot be excluded. Based on the heterogeneity of methods and results between studies, further validation is fundamental before conclusions can be drawn. The recognition of fluid biomarkers reflecting α-syn pathology is urgent [33] and may help not only in diagnosis but also in developing effective treatment and prevention strategies.

Additionally, one fact to be optimistic about is that recent studies support that proteins expressed during the inflammatory changes in synucleinopathies, such asCSF APR proteins, may be proven to be potential biomarkers of clinical diagnostic utility [34].

## Figures and Tables

**Table 1:** Results of recent studies on classical CSF biomarkers in DLB.

Reference	Reduced Aβ_42_	Increased τ_P-181_	Typical AD Profile	Age	Gender (F/M)	MMSE	Percentage
Bousiges et al., 2016 [8]	+	−	−	68.8	6/14	21	N/R
Van Steenoven et al., 2016 [10]	+	+	+	71.1 ^a^	125/248 ^a^	22 ^a^	25% ^a^
Van Steenoven et al., 2019 [11]	+	N/R	N/R	68	7/65	23	N/R
Abdelnour et al., 2016 [12]	+	+	+	74.22 ^a^	16/16 ^a^	21.09 ^a^	N/R
Paraskevas et al., 2019 [13]	+	+	+	75.4 ^a^	6/9 ^a^	12.7 ^a^	39.5% ^a^
Schoonenboom et al., 2012 [14]	+	+	+	69	12/40	23	47% ^a^

CSF: Cerebrospinal Fluid; DLB: Dementia with Lewy Bodies; Aβ_42_ Amyloid beta peptide with 42 amino acids; τ_P-181_: tau protein phosphorylated at threonine 181; AD: Alzheimer’s disease. + Observed; − not observed; ^a^: concerns the populations with typical AD profile; NR: Not Reported.

## Data Availability

Not applicable.
